# Denatured State Structural Property Determines Protein Stabilization by Macromolecular Crowding: A Thermodynamic and Structural Approach

**DOI:** 10.1371/journal.pone.0078936

**Published:** 2013-11-12

**Authors:** Shruti Mittal, Laishram Rajendrakumar Singh

**Affiliations:** Dr. B. R. Ambedkar Center for Biomedical Research, University of Delhi, Delhi, India; University of Hyderabad, India

## Abstract

Understanding of protein structure and stability gained to date has been acquired through investigations made under dilute conditions where total macromolecular concentration never surpasses 10 g l^−1^. However, biological macromolecules are known to evolve and function under crowded intracellular environments that comprises of proteins, nucleic acids, ribosomes and carbohydrates etc. Crowded environment is known to result in altered biological properties including thermodynamic, structural and functional aspect of macromolecules as compared to the macromolecules present in our commonly used experimental dilute buffers (for example, Tris HCl or phosphate buffer). In this study, we have investigated the thermodynamic and structural consequences of synthetic crowding agent (Ficoll 70) on three different proteins (Ribonuclease-A, lysozyme and holo α-lactalbumin) at different pH values. We report here that the effect of crowding is protein dependent in terms of protein thermal stability and structure. We also observed that the structural characteristics of the denatured state determines if crowding will have an effect or not on the protein stability.

## Introduction

Most solvent environments used traditionally to study basic biological processes including protein folding, enzyme activity, structural allostery etc were performed under highly dilute conditions as compared to the highly crowded intracellular environment wherein proteins perform their biological functions [1], [2]. Indeed, the cell interior is known to be densely populated due to the presence of soluble and insoluble macromolecules (proteins, nucleic acids, ribosomes and carbohydrates etc) [1], [3], [4], which together make the intracellular environment “crowded” or “volume-occupied” rather than “concentrated” [3], [5], [6], [7]. These macromolecules collectively occupy ∼10–40% (a substantial fraction of the intracellular space) of the total fluid volume, restricting the volume available to other macromolecules present. Crowded environment therefore, results in altered biological processes including thermodynamic, functional and structural properties of macromolecules as compared to the macromolecules present in dilute buffers. Thus, it is important to perform studies under conditions that mimic the environment of the crowded intracellular milieu to have a more realistic insight of the *in vivo* scenario. Nowadays availability of synthetic crowding agents (like Ficoll, Dextran etc) has made it possible to investigate the effect of macromolecular crowding on the properties of macromolecules.

Effect of macromolecular crowding on protein structure and stability has been widely investigated [8], [9], [10], [11], [12]. However, majority of the earlier studies investigating the effect of crowding on protein structure and stability were largely focused on intrinsically disordered proteins (IDPs) or under denaturing (and hence aggregating) experimental conditions [13], [14], [15], [16], [17], [18], [19], because it is commonly believed that the phenomenon of crowding mainly acts on the less compact, unfolded state rather than the more compact native state [5], [20], [21], [22]. Very few data are available in the literature about the effect of crowding on native state of proteins. Observations made so far on some proteins suggest that crowding increases native state structure, stability [8], [10], [11], [23], [24], and even induce shape changes in certain proteins [25], [26]. However, recently macromolecular crowding has also been shown to have opposite influence on certain proteins i.e., it induces structural and thermodynamic destabilization [27], [28], [29]. Therefore, the possible effect of macromolecular crowding on the native state structure and stability of proteins has not been properly understood. In the present communication using thermodynamic and structural approach, we investigated the effect of macromolecular crowding (using Ficoll 70) on the native state of three proteins (Ribonuclease-A, lysozyme and holo α-lactalbumin) having different physico-chemical properties. We discovered that the effect of macromolecular crowding is protein dependent in terms of native state structure and stability. We also observed that the structural characteristic of the denatured state of the protein determines whether crowding will have a stabilizing effect or not on protein stability.

## Materials and Methods

### 2.1. Materials

Commercially lyophilized preparations of Ribonuclease-A (RNase-A; from bovine pancreas), lysozyme (from chicken egg white), and holo α-Lactalbumin (α-LA; from bovine milk) were purchased from Sigma Chemical Co. Ficoll 70, *N*-Acetyl-L-tryptophanamide (NATA) and sodium salt of cacodylic acid were also obtained from Sigma Chemical Co. Potassium chloride and sodium acetate were obtained from Merck. Guanidinium chloride (GdmCl) was the ultrapure sample from MP Biomedicals. These and other chemicals, which were of analytical grade, were used without further purification.

### 2.2. Analytical Procedures

RNase-A, lysozyme and α-LA solutions were dialyzed extensively against 0.1 M KCl at pH 7.0 in cold (∼4°C). Protein stock solutions were filtered using 0.22-µm millipore filter paper. All the proteins gave a single band during polyacrylamide gel electrophoresis. Concentration of the protein solutions was determined experimentally using *ε*, the molar absorption coefficient values of 9800 M^−1^ cm^−1^ at 277.5 nm for RNase-A [30], 39000 M^−1^ cm^−1^ at 280 nm for Lysozyme [31], and 29210 M^−1^ cm^−1^ at 280 nm for α-LA [32]. The concentration of GdmCl stock solution was determined by refractive index measurements [33]. All solutions for optical measurements were prepared in the desired degassed buffer. For various pH ranges, the buffers used were 0.05 M acetate buffer (pH range 4.0–5.0) and 0.05 M cacodylic acid buffer containing 0.1 M KCl (pH range 6.0–7.0). Special care was taken to mix all solutions due to the high viscosity of Ficoll 70. Since pH of the protein solution may change on the addition of co-solvents, pH of each solution was also measured after the denaturation experiments. It should, however, be noted that no corrections were made for the possible effect of co-solvents on the observed pH of protein solutions.

### 2.3. Thermal Denaturation Studies

Thermal denaturation studies were carried out in a Jasco V-660 UV/Visible spectrophotometer equipped with a Peltier-type temperature controller at a heating rate of 1°C per minute. This scan rate was found to provide adequate time for equilibration. Each sample was heated from 20 to 85°C. The change in absorbance with increasing temperature was followed at 287 nm for RNase-A, 300 nm for lysozyme and 295 nm for α-LA. About 650 data points of each transition curve were collected. Measurements were repeated three times. After denaturation, the protein sample was immediately cooled down to measure reversibility of the reaction. Each heat-induced transition curve was analyzed for *T*_m_ (midpoint of denaturation) and Δ*H*_m_ (denaturational enthalpy change at *T*_m_) using a non-linear least-squares method according to the relation (Equation 1),
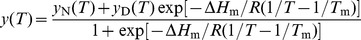
(1)where *y*(*T*) is the optical property at temperature *T* (Kelvin), *y*_N_(*T*) and *y*_D_(*T*) are the optical properties of the native and denatured protein molecules at *T* K, respectively, and R is the gas constant. In the analysis of the transition curve, it was assumed that a parabolic function describes the dependence of the optical properties of the native and denatured protein molecules (i.e. *y*_N_(*T*) = a_N_+b_N_ *T*+c_N_ *T* ^2^ and *y*_D_(*T*) = a_D_+b_D_ *T*+c_D_ *T* ^2^, where a_N_, b_N_, c_N_, a_D_, b_D_, and c_D_ are temperature-independent coefficients) [34]. A plot of Δ*H*_m_ versus *T*_m_ at each concentration of Ficoll 70 gave the value of Δ*C*_p_, the change in heat capacity at constant pressure. The value of Δ*G*_D_ at any temperature *T*, Δ*G*_D_(*T*), was estimated with the help of the Gibbs-Helmholtz equation (Equation 2) with values of Δ*H*_m_, *T*_m_ and Δ*C*_p_.
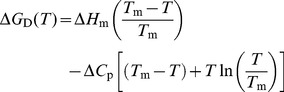
(2)


### 2.4. Circular Dichroism (CD) Measurements

CD measurements were made in a Jasco J-810 spectropolarimeter equipped with a Peltier-type temperature controller with six accumulations. Protein concentration used for the CD measurements was 0.5 g/l. Cells of 0.1 and 1.0 cm path length were used for the measurements of the far- and near-UV spectra, respectively. Necessary blanks were subtracted. The CD instrument was routinely calibrated with D-10-camphorsulfonic acid.

### 2.5. Fluorescence Measurements

Fluorescence spectra were measured in a PerkinElmer LS 55 Spectrofluorimeter in a 3 mm quartz cell, with both excitation and emission slits set at 10 nm. Protein concentration for all the experiments was 2 µM for lysozyme; 5 µM for RNase-A and α-LA. The concentration of NATA was 2 µM. For RNase-A, the excitation wavelength was 268 nm, while the emission spectra were recorded from 290–400 nm. Lysozyme, α-LA and NATA were excited at 280 nm and the emission spectra were recorded in the wavelength region 300–500 nm.

## Results

RNase-A, lysozyme and α-LA were selected because these proteins have been extensively characterized in terms of their chemical and thermal unfolding behaviours in dilute solutions. These proteins vary in their hydrophobicity index, ranging from 780 for RNase-A, 890 for lysozyme to 1050 for α-LA; and pI values are in the range of 9.5 for RNase-A, 10.7 for lysozyme to 5.0 for α-LA. The crowding agent selected for this study was Ficoll 70 for the following properties; it is a compact and highly cross-linked and branched copolymer of sucrose and epichlorohydrin that behaves like a semirigid sphere. It is inert, polar and does not interact with proteins, thus making it widely accepted as a test system for isolating effects of macromolecular crowding created by globular macromolecules found in the biological setting where proteins normally perform their function [35], [36], [37].

**Figure 1:**
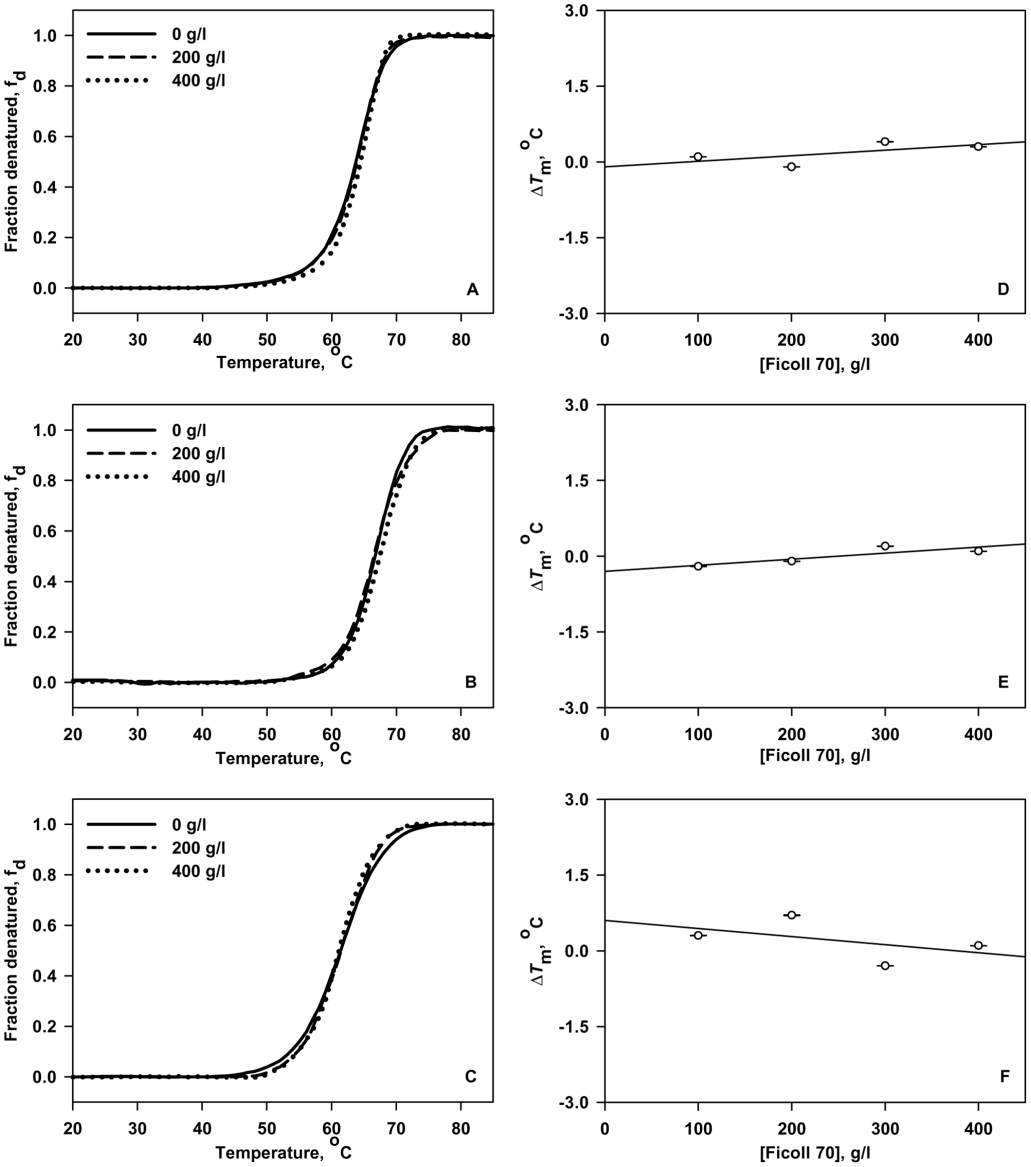
Representative thermal denaturation profiles of RNase-A, lysozyme and α-LA at pH 7.0. Denaturation curves of RNase-A (A), lysozyme (B) and α-LA (C) in the absence and presence of various Ficoll 70 concentrations at pH 7.0 (left panel). In order to maintain clarity, only the transition curves obtained in the presence of 200 and 400 g/l of Ficoll 70 are shown. Plots of Δ*T*_m_ versus [Ficoll 70] for RNase-A (D), lysozyme (E) and α-LA (F) at the indicated pH (right panel).

**Figure 2:**
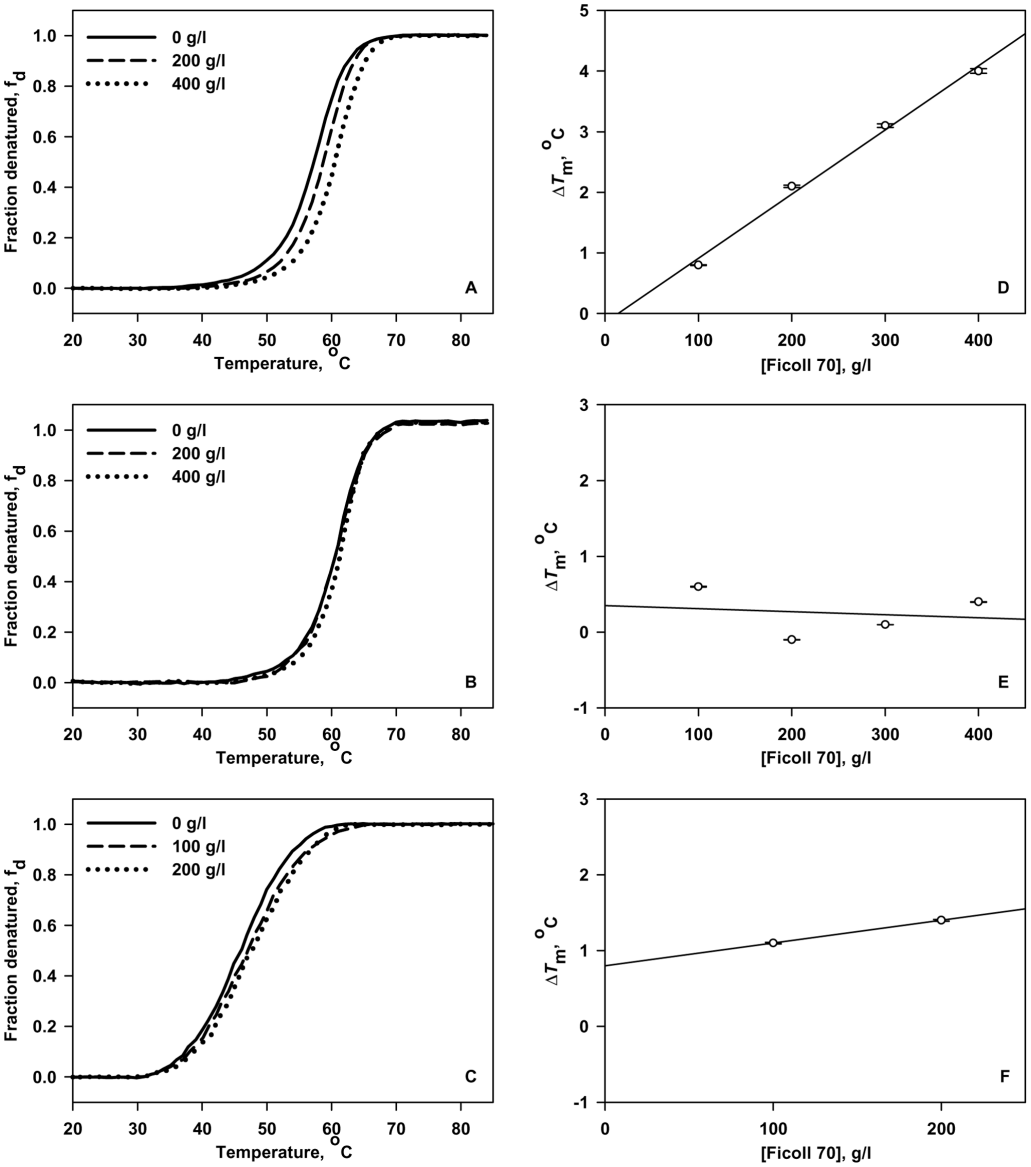
Representative thermal denaturation profiles of RNase-A, lysozyme and α-LA at pH 4.0. Denaturation curves of RNase-A (A), lysozyme (B) and α-LA (C) in the absence and presence of various Ficoll 70 concentrations at pH 4.0 (left panel). In order to maintain clarity, only the transition curves obtained in the presence of 200 and 400 g/l of Ficoll 70 are shown. In case of α-LA, curves for 100 g/l and 200 g/l Ficoll 70 has been shown as we could not obtain transition curves at higher [Ficoll 70] (see “Results” for detail). Plots of Δ*T*_m_ versus [Ficoll 70] for RNase-A (D), lysozyme (E) and α-LA (F) at the indicated pH (right panel).

Heat-induced denaturation studies of the three proteins were carried out in the presence of different Ficoll 70 concentrations (0, 100, 200, 300 and 400 g/l) at different pH values (7.0, 6.0, 5.0 and 4.0) by following the changes in absorbance at 287 nm for RNase-A, 300 nm for lysozyme and 295 nm for α-LA as a function of temperature. We could not go beyond pH 4.0 as Ficoll becomes unstable or gets hydrolyzed below pH 3.0 (see webpage of GE Healthcare Life Sciences). Denaturation of each protein was reversible in the entire range of [Ficoll 70], the molar concentration of Ficoll 70. However, it is also important to note that we observed visible precipitation of α-LA at pH 5.0 and below in the presence of Ficoll 70 concentration greater than 300 g/l. Therefore, we could not obtain reversible heat-induced transition curves under these experimental conditions in case of α-LA. Figure 1 (left panel) and Figure 2 (left panel) shows representative heat-induced denaturation profiles of RNase-A, lysozyme and α-LA at pH 7.0 and pH 4.0 respectively. In the case of lysozyme, complete transition curves could not be obtained in the measurable temperature range at the given pH values. In order to bring down transition curves in the measurable temperature range, 1.5 M GdmCl was added to the samples. Therefore, the transition curves shown in Figures 1 (left panel) and 2 (left panel) for lysozyme are the curves obtained in the presence of GdmCl. Each denaturation curve of a protein at a given [Ficoll 70] was analyzed for Δ*H*_m_ and *T*_m_ using a nonlinear least-squares method that involves fitting the entire data of the transition curve to equation (1) with all eight free parameters (a_N_, b_N_, c_N_, a_D_, b_D_, c_D_, Δ*H*_m_ and *T*_m_). Table 1 shows values of Δ*H*_m_ and *T*_m_ of all the three proteins in the absence and presence of different [Ficoll 70] at all the pH values. Figure 1 (right panel) and Figure 2 (right panel) shows plots of Δ*T*_m_ versus [Ficoll 70] at pH 7.0 and pH 4.0 respectively for all the three proteins; Δ*T*_m_ is the difference between *T*_m_ values in the presence and absence of different [Ficoll 70]. It is seen in Figure 1 (right panel) and Table 1 that the *T*_m_ of all the proteins remains unperturbed in the presence of Ficoll 70 at physiological pH. However, *T*_m_ of the proteins at pH 4.0 in the presence of Ficoll 70 was differently affected.

**Table 1:** Thermodynamic parameters of RNase-A, Lysozyme and α-LA in the presence of Ficoll 70a,b.

RNase-A													
	pH 7.0			pH 6.0			pH 5.0			pH 4.0			
[Ficoll 70]	*T*_m_	Δ*H*_m_	Δ*G*_D_^o^	*T*_m_	Δ*H*_m_	Δ*G*_D_^o^	*T*_m_	Δ*H*_m_	Δ*G*_D_^o^	*T*_m_	Δ*H*_m_	Δ*G*_D_^o^	Δ*C*_p_
0	63.7	106	9.08	61.5	104	8.57	60.2	101	8.08	56.8	97	7.13	1.34
100	63.8	108	8.97	61.6	106	8.49	61.1	104	8.22	57.6	99	7.28	1.49
200	63.6	111	9.14	61.8	105	8.25	61.9	106	8.38	58.9	103	7.61	1.56
300	64.1	114	9.04	62.1	112	8.62	62.8	110	8.46	59.9	106	7.82	1.80
400	64.0	117	9.12	62.5	114	8.64	63.4	115	8.84	60.8	111	8.15	1.90
**Lysozyme**													
	**pH 7.0**			**pH 6.0**			**pH 5.0**			**pH 4.0**			
**[Ficoll 70]**	***T*** **_m_**	**Δ** ***H*** **_m_**	**Δ** ***G*** **_D_^o^**	***T*** **_m_**	**Δ** ***H*** **_m_**	**Δ** ***G*** **_D_^o^**	***T*** **_m_**	**Δ** ***H*** **_m_**	**Δ** ***G*** **_D_^o^**	***T*** **_m_**	**Δ** ***H*** **_m_**	**Δ** ***G*** **_D_^o^**	**Δ** ***C*** **_p_**
0	67.0	100	7.85	65.2	94	7.04	63.0	93	6.81	61.0	89	6.25	1.66
100	66.8	100	7.71	65.6	95	7.05	63.2	93	6.70	61.6	90	6.29	1.71
200	66.9	102	7.77	65.0	96	6.97	62.8	93	6.53	60.9	91	6.22	1.78
300	67.2	103	7.69	65.3	99	7.14	63.2	94	6.48	61.1	92	6.17	1.86
400	67.1	103	7.61	65.9	100	7.20	63.1	95	6.52	61.4	92	6.12	1.89
**α-LA**													
	**pH 7.0**			**pH 6.0**			**pH 5.0**			**pH 4.0**			
**[Ficoll 70]**	***T*** **_m_**	**Δ** ***H*** **_m_**	**Δ** ***G*** **_D_^o^**	***T*** **_m_**	**Δ** ***H*** **_m_**	**Δ** ***G*** **_D_^o^**	***T*** **_m_**	**Δ** ***H*** **_m_**	**Δ** ***G*** **_D_^o^**	***T*** **_m_**	**Δ** ***H*** **_m_**	**Δ** ***G*** **_D_^o^**	**Δ** ***C*** **_p_**
0	60.5	64	4.05	64.1	72	5.02	62.0	70	4.74	45.9	46	2.03	1.41
100	60.8	67	4.24	63.8	74	5.08	62.3	73	4.93	47.0	49	2.22	1.48
200	61.2	69	4.30	64.0	76	5.20	62.3	75	4.99	47.3	51	2.30	1.53
300	60.2	75	N.D.	63.3	77	N.D.	N.D.	N.D.	N.D.	N.D.	N.D.	N.D.	N.D.
400	60.6	79	N.D.	64.4	80	N.D.	N.D.	N.D.	N.D.	N.D.	N.D.	N.D.	N.D.

a The units of [Ficoll 70], *T*_m_, Δ*H*_m_, Δ*G*_D_^o^ and Δ*C*_p_ are g l^−1^, °C, kcal mol^−1^, kcal mol^−1^ and kcal mol^−1^ K^−1^, respectively.

b Errors in *T*_m_, Δ*H*_m_, Δ*G*_D_^o^ and Δ*C*_p_ are 0.2–1%, 3–6%, 6–9% and 5–7%, respectively.

N.D. Not determined.

Table 1 also shows Δ*C*_p_ values obtained from the analyses of the plots of Δ*H*_m_ versus *T*_m_ at 4 different pH values in the absence and presence of different [Ficoll 70]. Using the measured values of Δ*H*_m_, *T*_m_ and Δ*C*_p_, Δ*G*_D_^o^ values (the value of Δ*G*_D_ at 25°C) in the presence and absence of Ficoll 70 were also estimated using equation (2). The values of Δ*G*_D_^o^ estimated in such manner are presented in Table 1. It is seen in Table 1 that similar to the effect of Ficoll 70 on *T*_m_, the Δ*G*_D_^o^ values are also not significantly perturbed at physiological pH. Surprisingly, the Δ*G*_D_^o^ values of the protein are perturbed differently at pH 4.0. We, therefore conclude that protein stability (in terms of *T*_m_ and Δ*G*_D_^o^) at physiological conditions is not influenced by macromolecular crowding.

**Figure 3:**
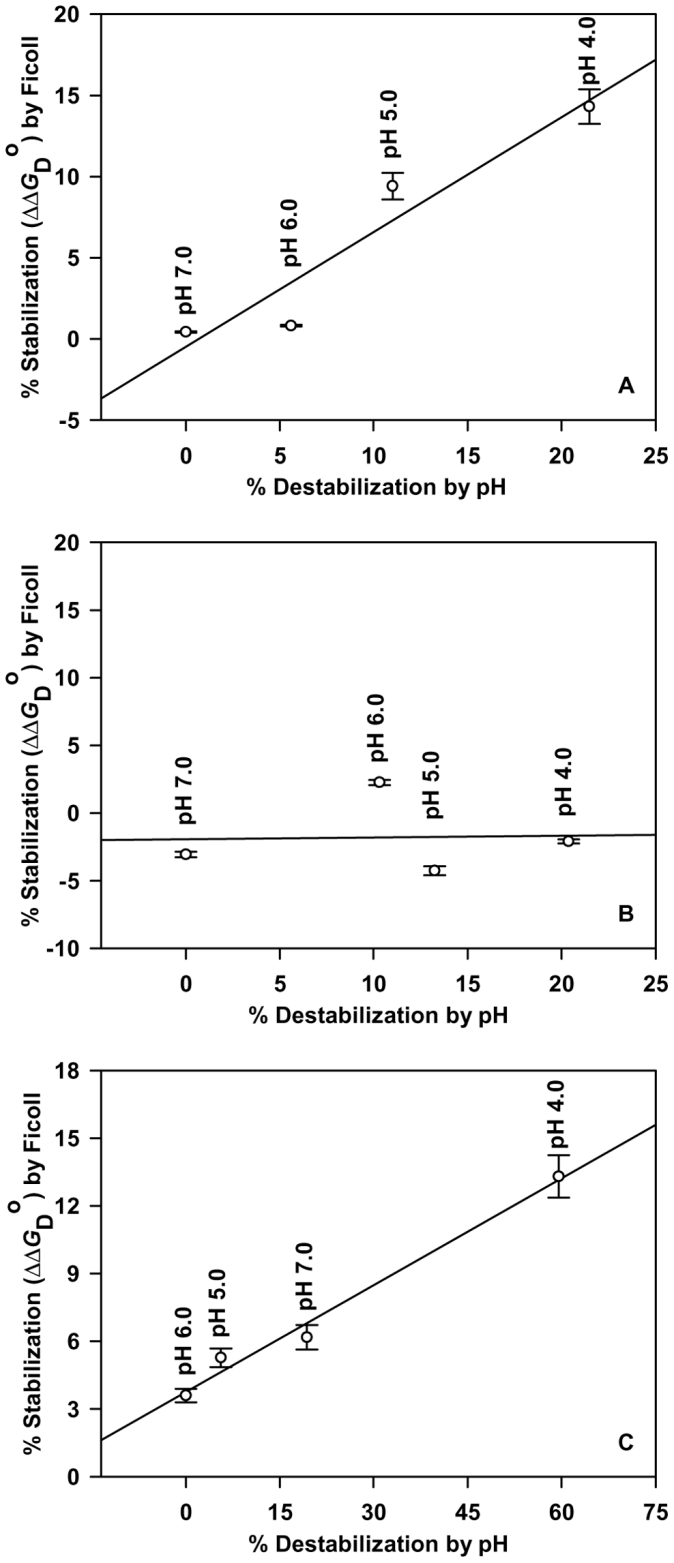
Plots of percent stabilization against percent destabilization. Plots of percent stabilization (%ΔΔ*G*_D_^o^) by Ficoll 70 versus percent destabilization by pH of RNase-A (A), lysozyme (B) and α-LA (C) in the presence of 400 g/l Ficoll 70 (200 g/l in case of α-LA). For the estimation of percent Ficoll 70 stabilization (%ΔΔ*G*_D_^o^), we show a model calculation. %ΔΔ*G*_D_^o^ of RNase-A in the presence of 400 g/l Ficoll 70 at pH 4.0 = 100×[(Δ*G*_D_^o^ in the presence of 400 g/l Ficoll 70–Δ*G*_D_^o^ in the absence of Ficoll 70)/Δ*G*_D_^o^ in the absence of Ficoll 70)] = 100×[(8.15–7.13)/7.13] = 14.31. Percent destabilization at any pH × with respect to the most stable pH condition (pH 6.0 for α-LA and pH 7.0 for RNase-A or lysozyme) is equal to 100×(Δ*G*_D_^(pH y)^–Δ*G*_D_^(pH x)^/Δ*G*_D_^(pH y)^). Thus, percent destabilization of RNase-A at pH 4.0 = 100×(9.08–7.13)/9.08 = 21.

To further investigate for the effect of pH on Ficoll 70 induced protein stabilization, we have plotted percent stabilization (%ΔΔ*G*_D_^o^) by Ficoll 70 versus percent destabilization by pH (Figure 3); %ΔΔ*G*_D_^o^ represents the difference between Δ*G*_D_^o^ values in the presence and absence of Ficoll 70 (400 g/l for RNase-A and lysozyme; 200 g/l for α-LA). For the estimation of percent stabilization (ΔΔ*G*_D_^o^) by Ficoll 70 and percent destabilization by pH, see legend to Figure 3. It is seen in Figure 3 that %ΔΔ*G*_D_^o^ values are increased due to the effect of Ficoll 70 on RNase-A and α-LA with increasing destabilization by pH. But, no such effect is observed in the case of lysozyme. It is important to note that in the case of lysozyme, %ΔΔ*G*_D_^o^ remains unperturbed even if we destabilize the protein by lowering the pH upto 4.0. The results indicate that crowding has different thermodynamic effects on the three different proteins depending on the properties of the proteins chosen.

**Figure 4:**
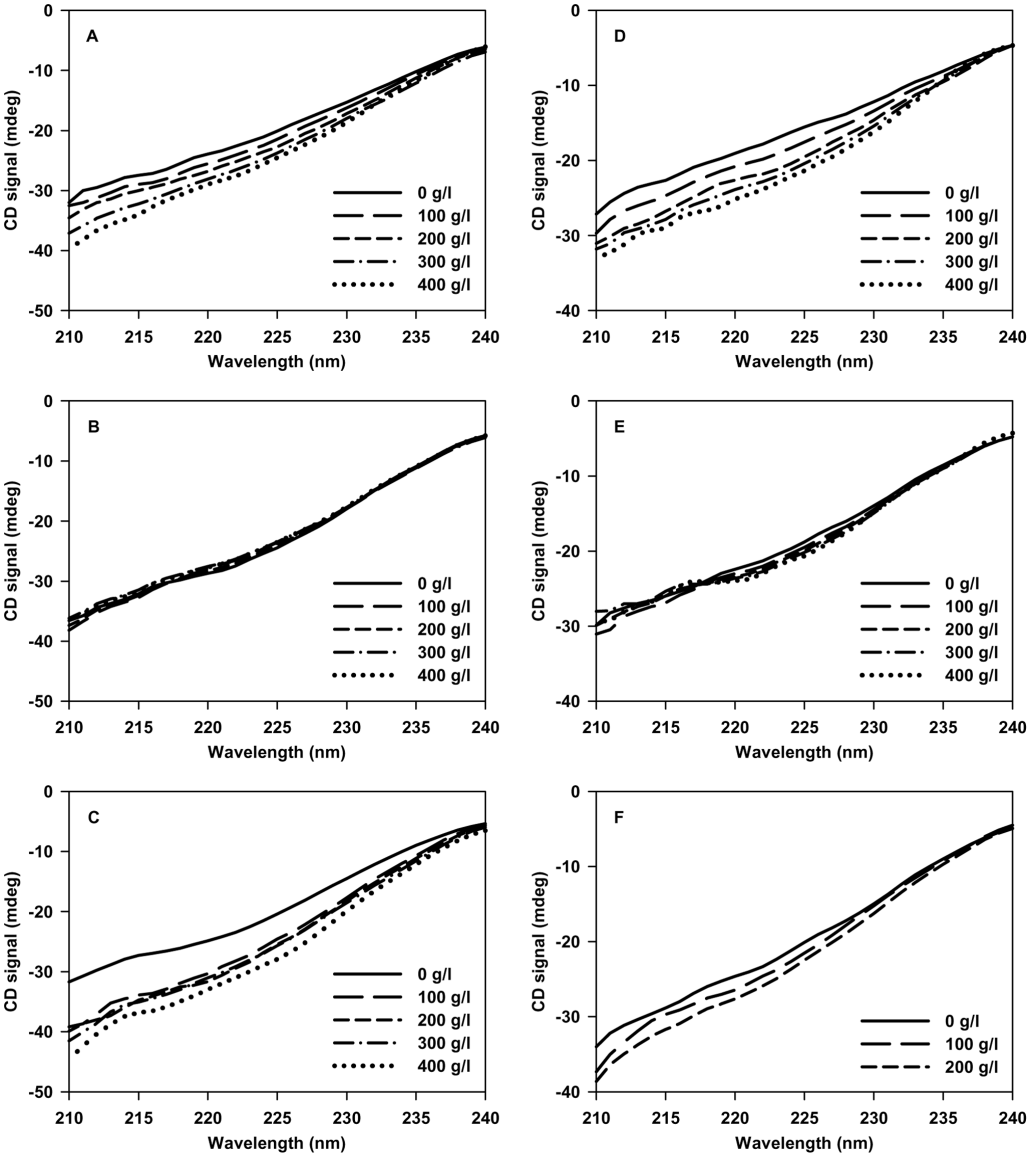
Effect of Ficoll 70 on the denatured states of proteins. Far-UV CD spectra of the denatured state of RNase-A (A), lysozyme (B) and α-LA (C) in the absence and presence of various Ficoll 70 concentrations at pH 7.0 (left panel). Far-UV CD spectra of the denatured state of RNase-A (D), lysozyme (E) and α-LA (F) in the absence and presence of various Ficoll 70 concentrations at pH 4.0 (right panel). The far-UV CD of lysozyme was measured in the absence of GdmCl.

**Figure 5:**
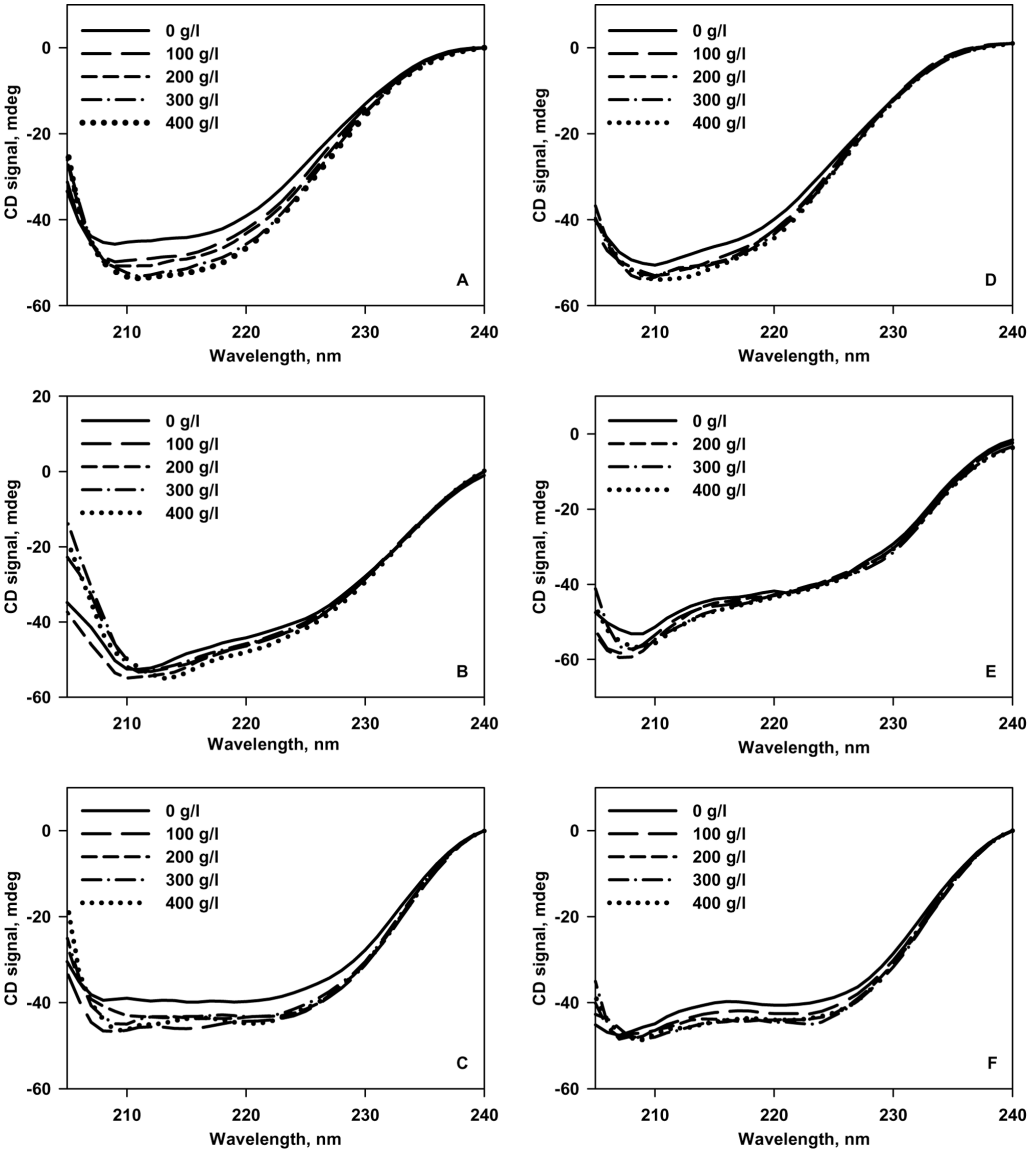
Effect of Ficoll 70 on the secondary structure of the native states of proteins. Far-UV CD spectra (at 25°C) of the native states of RNase-A (A), lysozyme (B) and α-LA (C) in the absence and presence of various Ficoll 70 concentrations at pH 7.0 (left panel). Far-UV CD spectra (at 25°C) of the native states of RNase-A (D), lysozyme (E) and α-LA (F) in the absence and presence of various Ficoll 70 concentrations at pH 4.0 (right panel).

**Figure 6:**
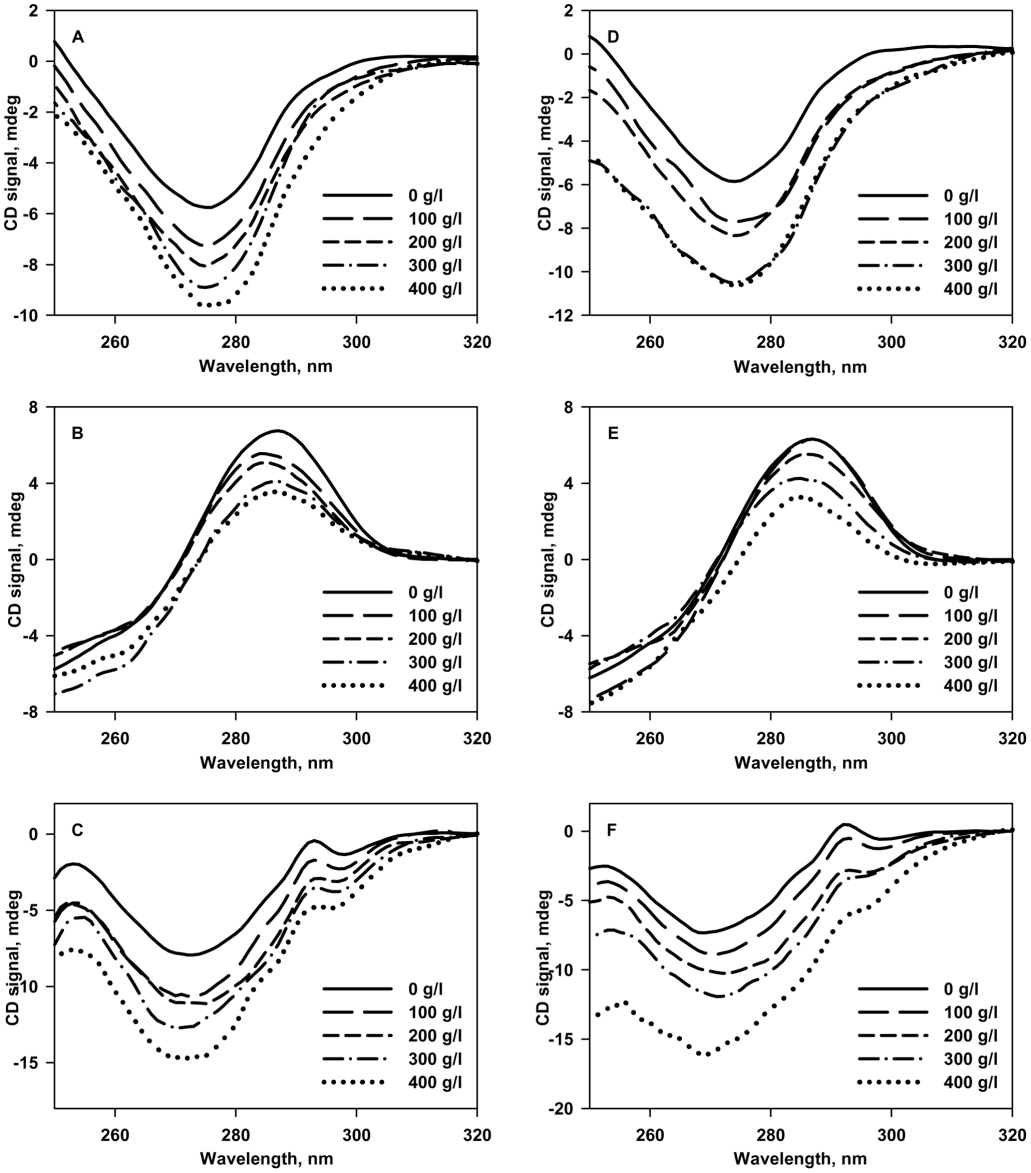
Effect of Ficoll 70 on the tertiary structure of the native state of proteins. Near-UV CD spectra (at 25°C) of the native states of RNase-A (A), lysozyme (B) and α-LA (C) in the absence and presence of various Ficoll 70 concentrations at pH 7.0 (left panel). Near-UV CD spectra (at 25°C) of the native states of RNase-A (D), lysozyme (E) and α-LA (F) in the absence and presence of various Ficoll 70 concentrations at pH 4.0 (right panel).

**Figure 7:**
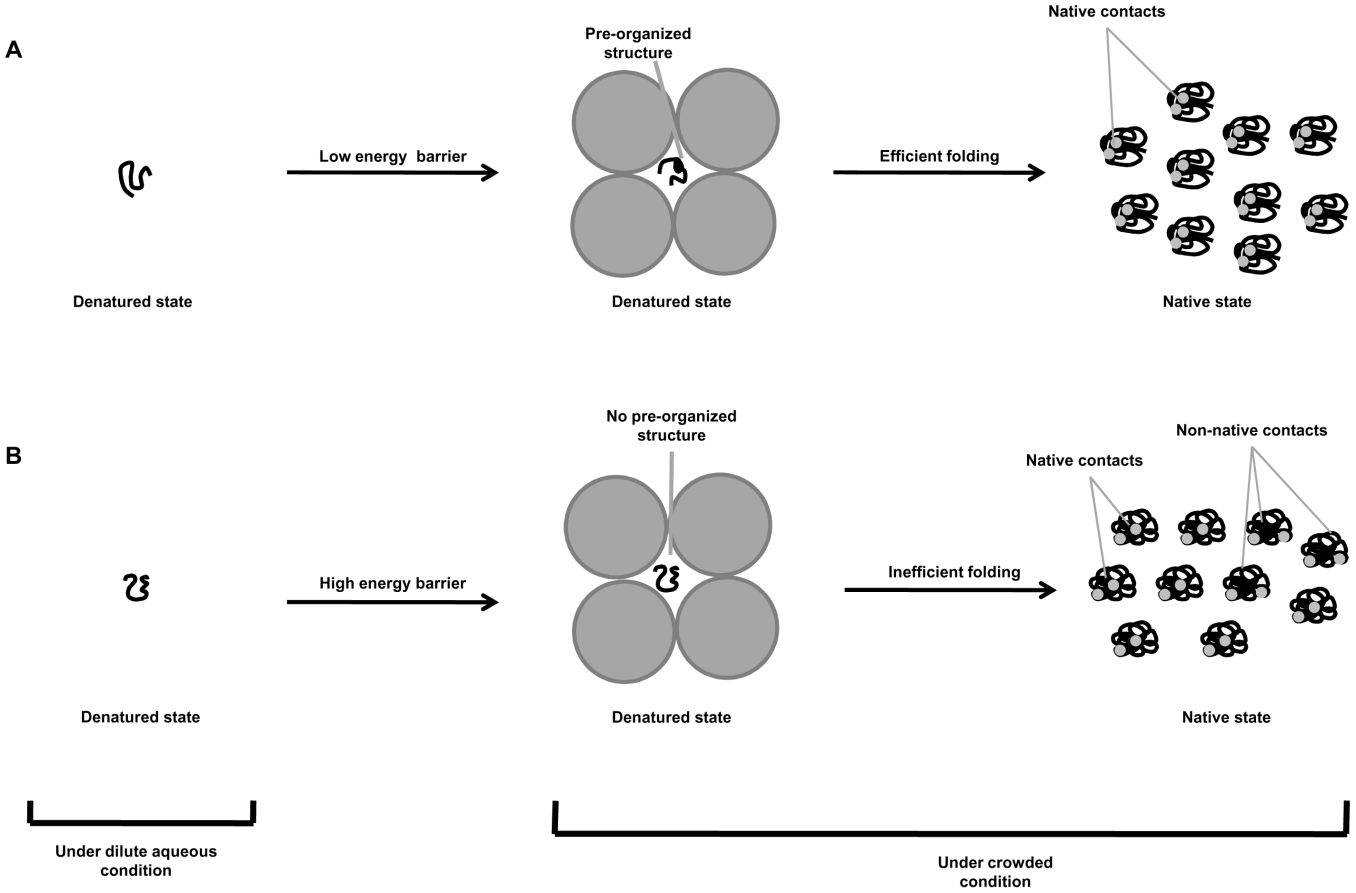
A schematic representation of the effect of macromolecular crowding on different denatured polypeptides. (A) Efficient folding of polypeptides due to formation of a preorganized denatured state under crowded conditions; (B) Inefficient folding of polypeptides due to lack of preorganized structure formation. Solid circles represent crowder molecules.

The effect of crowding on the structural properties of denatured state (D-state) of the proteins was then investigated using far-UV CD at pH 7.0 and pH 4.0. Figure 4 shows the effect of increasing [Ficoll 70] on the structural properties of the D-state of all the 3 proteins. The CD spectra of all the three proteins under dilute conditions are reminiscent of an unfolded protein. Addition of Ficoll 70 reflects a gain in secondary structure in the case of RNase-A and α-LA at both the pH values. In contrast, crowding is observed not to perturb the D-state structure significantly in case of lysozyme at both pH values suggesting that crowding affects the D-state structure of different proteins differently. We further examined for the possible alteration in the native state structure of proteins due to the presence of Ficoll 70 at pH 7.0 and 4.0. For this, we measured far-UV CD (Figure 5), near-UV CD (Figure 6) and tyr/trp fluorescence (Figure S1). Left panels represent measurements at pH 7.0, while right panels represent measurements at pH 4.0 for Figures 5, 6 and 7. It is seen in these figures that Ficoll 70 has different effects on lysozyme as compared to RNase-A and α-LA in terms of secondary and tertiary structures at both the pH values. The structural measurements therefore, indicate that crowding has different effects on the N-state structural properties of different proteins. Control experiments with NATA were also performed to ensure that the crowding effects observed on the fluorescence spectral properties of the three proteins are not simply a solvent effect (Figure S2).

## Discussion

Effect of Ficoll 70 on the stability of three different proteins (RNase-A, lysozyme and α-LA) was determined by measuring the heat-induced denaturation of the proteins, for which change in Δ*A*_λ_, the difference in absorbance at the wavelength λ; Δ*A*_287_ for RNase-A, Δ*A*_300_ for lysozyme and Δ*A*_295_ for α-LA was monitored. Analysis of a thermal denaturation curve according to equation (1) assumes that the denaturation follows a two-state mechanism. It is indeed true for all the three proteins in the absence of Ficoll 70 [38], [39]. To check whether the two-state assumption is valid in the presence of Ficoll 70, the heat-induced denaturation of RNase-A, lysozyme and α-LA in the presence of 400 g/l Ficoll 70 (except at pH 5.0 and below for α-LA) were monitored by two different optical techniques, namely, mean residue ellipticity at 222 nm ([*θ*]_222_) which measures the change in the peptide backbone conformation (denaturation curves not shown), and change in absorbance (Δ*A*_λ_) (representative curves at two different pH values are shown in Figures 1 and 2; left panel), which measures the change in the tyr/trp environment. The concentration of Ficoll 70 used for α-LA at pH 5.0 and below were 200 g/l as the protein got precipitated in the presence of 400 g/l Ficoll 70 at these pH values. We compared Δ*H*_m_ and *T*_m_ values obtained from absorbance measurements with those obtained from [*θ*]_222_ measurements. Both measurements gave, within experimental errors, identical values of these thermodynamic parameters (Table S1). Thus, a two-state assumption for thermal denaturation of RNase-A, lysozyme and α-LA in the presence of Ficoll 70, is valid.

Because the thermodynamic parameters of proteins reported here are obtained by an indirect method based on equilibrium denaturation, it is, therefore, necessary to validate them against those obtained directly by a thermodynamic method. Indeed Δ*H*_m_, *T*_m_ and Δ*C*_p_ of proteins in the absence of Ficoll 70 (see Table 1) are in excellent agreement with those obtained from differential scanning calorimetry measurements [38], [40]; for example, the calorimetric values of Δ*C*_p_ are 1.22, 1.60 and 1.36 kcal mol^−1^ K^−1^ for RNase-A, lysozyme and α-LA respectively. This agreement led us to believe that our measurements of transition curves and our analysis of these curves for thermodynamic parameters are authentic and accurate.

At a constant pH and Ficoll 70 concentration, Δ*G*_D_^o^ was estimated using equation (2) with known values of Δ*H*_m_, *T*_m_ and Δ*C*_p_. However, this estimation requires a large extrapolation. Hence, a large error may be associated with Δ*G*_D_^o^ determination due to errors in the estimations of Δ*H*_m_, *T*_m_ and Δ*C*_p_. We have used Becktel and Schellman’s procedure [41] to determine the maximum and minimum errors associated with the Δ*G*_D_^o^ determination at a given solvent condition. This procedure involves the estimation of Δ*G*_D_^o^ of proteins using the maximum and minimum fitting parameter errors of Δ*H*_m_ and Δ*C*_p_ (one with maximum error in Δ*H*_m_ and minimum error in Δ*C*_p_ and the other with minimum error in Δ*H*_m_ and maximum error in Δ*C*_p_) obtained from the analysis of individual denaturation curves to yield two different Δ*G*_D_^o^ values (one minimum and one maximum). Because there were three independent measurements of Δ*H*_m_ and *T*_m_ of a protein at the given pH and [Ficoll 70], we obtained six values of Δ*G*_D_^o^ (three maximum and three minimum values). All of these six values were used to determine the average Δ*G*_D_^o^ and the mean error. It was observed that the mean error associated with the Δ*G*_D_^o^ estimation was in the range 6–9% for all proteins.

It is seen in Table 1 that Ficoll 70 has no significant effect on the thermodynamic stability of all the three proteins at pH 7.0 revealing that addition of Ficoll 70 to protein solutions does not alter either *T*_m_ (see Figure 1) or Δ*G*_D_^o^ of the proteins. Currently, the influence of macromolecular crowding on protein stability is explained based on two types of interactions – hard core repulsion (or volume exclusion) and soft interactions (non-specific chemical interactions) [24]. Hard-core repulsions decrease the space available to the protein under study thereby increasing protein stability. On the other hand, soft interactions can be attractive (destabilizing) or repulsive (stabilizing). Attractive interactions are destabilizing while repulsive interactions are stabilizing [24]. Therefore, the observed effect (no change in Δ*G*_D_^o^ in the presence of crowding agent) on the three proteins used in this study might be due to a perfect balance between the stabilizing and destabilizing interactions between the protein and crowder molecules. Interestingly, it has previously been shown that stabilizing hard-core repulsions can be completely offset by destabilizing soft interactions between the test protein and the crowder molecules [42]. In contrast to the present finding, studies performed earlier showed that macromolecular crowding increases thermodynamic stability of proteins. However, most of these earlier studies were largely confined to denaturing solvent conditions [14], [15], [17], [18], [19], [43]. Interestingly, the proteins used in this study were relatively more stable than those reported earlier for the effect of macromolecular crowding. We therefore speculated that Ficoll 70 will have stabilizing effect on the three proteins under conditions that populate large number of unfolded molecules. In this spirit, we intentionally destabilize the proteins by lowering pH. It is seen in Figure 2 that Ficoll 70 has a stabilizing influence on RNase-A and α-LA at pH 4.0. Results shown in Figure 3a and 3c also indicate that greater the destabilization of RNase-A and α-LA, larger is the stabilizing effect of Ficoll 70 confirming that our hypothesis is indeed true. In addition, the observed precipitation of α-LA at higher concentrations of Ficoll 70 (300 g/l and above) at pH 5.0 and below might be due to availability of limited volume (as a consequence of excluded volume effect) to accommodate large amounts of unfolded molecules. However, the thermal denaturation profiles and the measured thermodynamic parameters of lysozyme at low pH values remains practically unchanged upon addition of Ficoll 70 relative to that in dilute aqueous solutions, suggesting that it experiences no or little, if any, crowding effect (Figure 2b and Table 1). It may be noted that the extent of destabilization of lysozyme at pH 4.0 relative to pH 7.0 is almost the same as that of RNase-A and therefore, increase in protein stability in the presence of Ficoll 70 is expected (Figure 3). Thus, the thermodynamic measurements on the three proteins revealed that the effect of macromolecular crowding is protein-crowder system dependent. It is speculated that due to difference in the chemical nature among proteins, different proteins interact differently with crowder because of different extents of hard and soft interactions, leading to perfect balance between the two interactions (e.g., in case of lysozyme) or overwhelming repulsive interactions (e.g., in case of RNase-A and α-LA) yielding a protein-crowder system dependent thermodynamic stability.

The pH-dependent effect of Ficoll 70 on the thermodynamic stability of both RNase-A and α-LA and no effect on lysozyme led us to believe that denatured state of the proteins might play a role towards the stabilizing influence of Ficoll 70. To verify this possibility, we heat denature each of the three proteins at 85°C and measured far-UV CD (a signature of secondary structural content) spectra of the proteins in the presence of different concentrations of Ficoll 70 (Figure 4) at pH 7.0 and pH 4.0. In agreement to our thermodynamic data, we observed that there is an increase in the residual structure of the heat denatured state upon addition of Ficoll 70 in case of both RNase-A and α-LA at both the pH values (Figures 4a, 4c, 4d and 4f) indicating that excluded volume effect can induce structure in the denatured state. Similar to our results, it has been shown that crowding induces structural enhancement in the unfolded/denatured state of various proteins [14], [15]. However, in case of lysozyme (Figure 4b and 4e) there is no increase in residual structure in the presence of Ficoll 70 suggesting that excluded volume effect could not induce structure in the heat denatured state. The observed effect of crowding on lysozyme is also in agreement with earlier report on other proteins including flavodoxin and VlsE [23]. We, therefore conclude that the concept of macromolecular crowding to induce protein folding by acting on floppy denatured state is not universally true. Interestingly, it has been demonstrated that the mutant of immunoglobulin G binding domain of protein L (ProtL) failed to fold inside *Escherichia coli* but is otherwise capable of folding in the presence of salts under dilute aqueous conditions [44]. Further investigations made so far to understand the effect of macromolecular crowding on various IDPs showed that crowding induces compaction without any structural gain in some IDPs [16], [45] while having no such effect on many other IDPs [44], [46], [47], [48]. In another development, crowding was also shown to induce structure only in the C-terminal half of FlgM, while the other part remains unstructured [13]. These evidences and our findings clearly indicate that crowding-induced effect on the different denatured states (or on different IDPs) might depend on the primary sequence (due to different structural characteristics) of the polypeptide chain.

At present we do not yet have any concrete explanation for the differential effect of crowding (Ficoll 70) on thermal stability of the three proteins at pH 4.0. However, it has been observed earlier that formation of pre-organized structure in the denatured state helps to nucleate folding process and also fasten up the rate of refolding process [49], [50]. The existence of such apparent pre-organized structures (or local structuring) are clearly seen in case of RNase-A and α-LA in the presence of Ficoll 70 but is absent in lysozyme. It might be possible that the energy barrier for the formation of pre-organized structure is quite higher for lysozyme than that of RNase-A and α-LA. Therefore, under crowded condition lysozyme must fold via random search while RNase-A and α-LA through a nucleation process resulting in inefficient folding in case of lysozyme and better folding in case of both RNase-A and α-LA (see Figure 7). Interestingly, refolding of oxidized lysozyme in the presence of Ficoll 70 decreases folding efficiency to ∼90% [9], while the refolding efficiency of RNase-A in the presence of crowding agent is increased by ∼20% relative to the dilute buffer [15]. Although many small globular proteins are known to fold spontaneously in a two state manner without the assistance of chaperones *in vitro* [51], [52], [53], our study indicates that some of such small globular proteins may require the assistance of chaperones to fold efficiently *in vivo*.

To further investigate the effect of macromolecular crowding on the native state structure, we have measured the far-UV CD, near-UV CD (a measure of gross tertiary interactions) and tyr/trp fluorescence at both pH 7.0 and 4.0. We found that Ficoll 70 alters the native state structure of the proteins differently at physiological pH (pH 7.0). In terms of secondary structure (Figure 5; left panel), Ficoll 70 does not have significant effect on lysozyme while having enhanced structural effect on both RNase-A and α-LA. In terms of tertiary interactions based on near-UV CD (Figure 6; left panel) and tyr/trp fluorescence (Figure S1; left panel), Ficoll 70 appears to have destabilizing influence on lysozyme while increasing the structural content of RNase-A and α-LA. It may be noted that changes in the spectral properties of the near-UV CD and fluorescence measurements might not be due to structural shifts but a representation of solvent effects. To verify this possibility, we measured NATA fluorescence in the absence and presence of 400 g/l Ficoll 70 (Figure S2). It has been observed that there was no significant change in the spectral properties of NATA under dilute and crowded conditions. The results led us to believe that the increase/decrease in *λ*_max_ of the proteins under crowded conditions is truely due to structural alterations (not a solvent effect). In agreement to our observations on lysozyme, many proteins have been reported to have increased conformational fluctuations in the presence of macromolecular crowding relative to the dilute aqueous buffers [27], [28], [29], [54], [55], [56]. Since, Ficoll 70 is found to alter the native state secondary and tertiary structures of the proteins, the insignificant effect of Ficoll 70 on the thermodynamic stability of the proteins at pH 7.0 might not be related to the structural alterations induced by Ficoll 70. It is speculated that Ficoll 70 introduces some new secondary or tertiary structure in RNase-A or α-LA that are co-operatively unfolded upon denaturation leading to no significant change in the thermal stability of the proteins. On the other hand, in the case of lysozyme, the tertiary interactions that are lost in the native state structure in the presence of Ficoll 70 might not contribute much to the thermodynamic stability of the protein, thereby leaving the thermodynamic stability unperturbed.

At pH 4.0 where Δ*G*_D_^o^ values of RNase-A and α-LA are increased in the presence of Ficoll 70 (Table 1), the tertiary interactions of both the proteins are increased although there is no significant effect on the secondary structures of the proteins (see Figures 5, 6; right panel). The results indicate that increase in tertiary structure of the proteins might have some contribution towards the increase in thermodynamic stability of the proteins by Ficoll 70 at pH 4.0. However, lysozyme shows destabilization in tertiary structure (as evidenced by the near-UV CD measurements) but the Δ*G*_D_^o^ remains unchanged suggesting that the native state structural changes might not be the direct consequence of having no significant effect on the thermodynamic stability of lysozyme. Therefore, it is highly unlikely that the native state structural increment will contribute towards the increase in Δ*G*_D_^o^ of proteins at pH 4.0 in case of RNase-A and α-LA. Taken together, the results indicate that (i) Structural consequences of macromolecular crowding are protein-crowder system dependent, (ii) The native state structural changes do not contribute to the thermodynamic stability of the proteins.

## Conclusion

In summary now we are sure of at least two things: (i) the structural and thermodynamic consequences of macromolecular crowding are protein-crowder system dependent, and (ii) the structural characteristic of the denatured state determines if macromolecular crowding will have an effect or not on the protein stability. It is well known that cells have various proteins having different structural and thermodynamic characteristics. Therefore, the effect of macromolecular crowding cannot be generalized based on studies available on certain proteins. It is speculated that IDPs, being natively unfolded may serve as a good model for denatured state of proteins and hence for crowding-induced protein folding. Effect of macromolecular crowding has been investigated on few IDPs, much still remains unexplored. Future research should focus on crowding-induced folding of many natively unfolded proteins and their structural regulation as it is possible that macromolecular crowding affects the structural regulation of natively unfolded proteins.

## Supporting Information

Figure S1 **Effect of Ficoll 70 on the intrinsic fluorescence of the native state of proteins.** Tyr/trp fluorescence (at 25°C) of RNase-A (A), lysozyme (B) and α-LA (C) in the absence and presence of 400 g/l Ficoll 70 at pH 7.0 (left panel). Tyr/trp fluorescence (at 25°C) of RNase-A (D), lysozyme (E) and α-LA (F) in the absence and presence of 400 g/l Ficoll 70 at pH 4.0 (right panel).
(TIF)

Figure S2 **Effect of Ficoll 70 on the fluorescence of NATA.** Intrinsic fluorescence of NATA in the absence and presence of 400 g/l Ficoll 70.
(TIF)

Table S1 (TIF)
