# Supplementary figures and images for: Denatured State Structural Property Determines Protein Stabilization by Macromolecular Crowding: A Thermodynamic and Structural Approach
Source: PLoS One. 2013 Nov 12;8(11):e78936. doi: 10.1371/journal.pone.0078936 (PMC3827121; doi:10.1371/journal.pone.0078936)

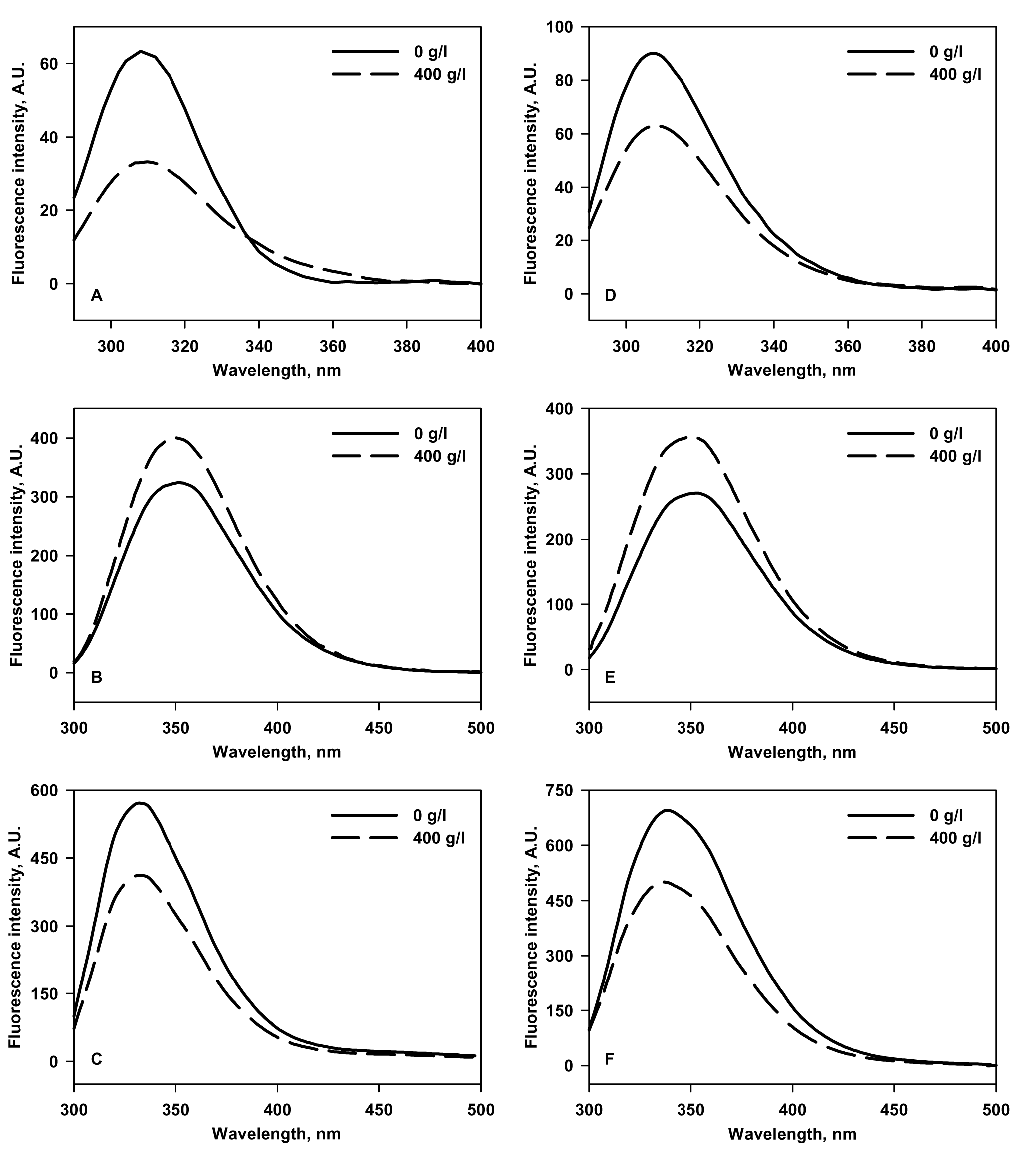

Supplement: Figure S1 — Effect of Ficoll 70 on the intrinsic fluorescence of the native state of proteins. Tyr/trp fluorescence (at 25°C) of RNase-A (A), lysozyme (B) and α-LA (C) in the absence and presence of 400 g/l Ficoll 70 at pH 7.0 (left panel). Tyr/trp fluorescence (at 25°C) of RNase-A (D), lysozyme (E) and α-LA (F) in the absence and presence of 400 g/l Ficoll 70 at pH 4.0 (right panel). (TIF) [file pone.0078936.s001.tif]

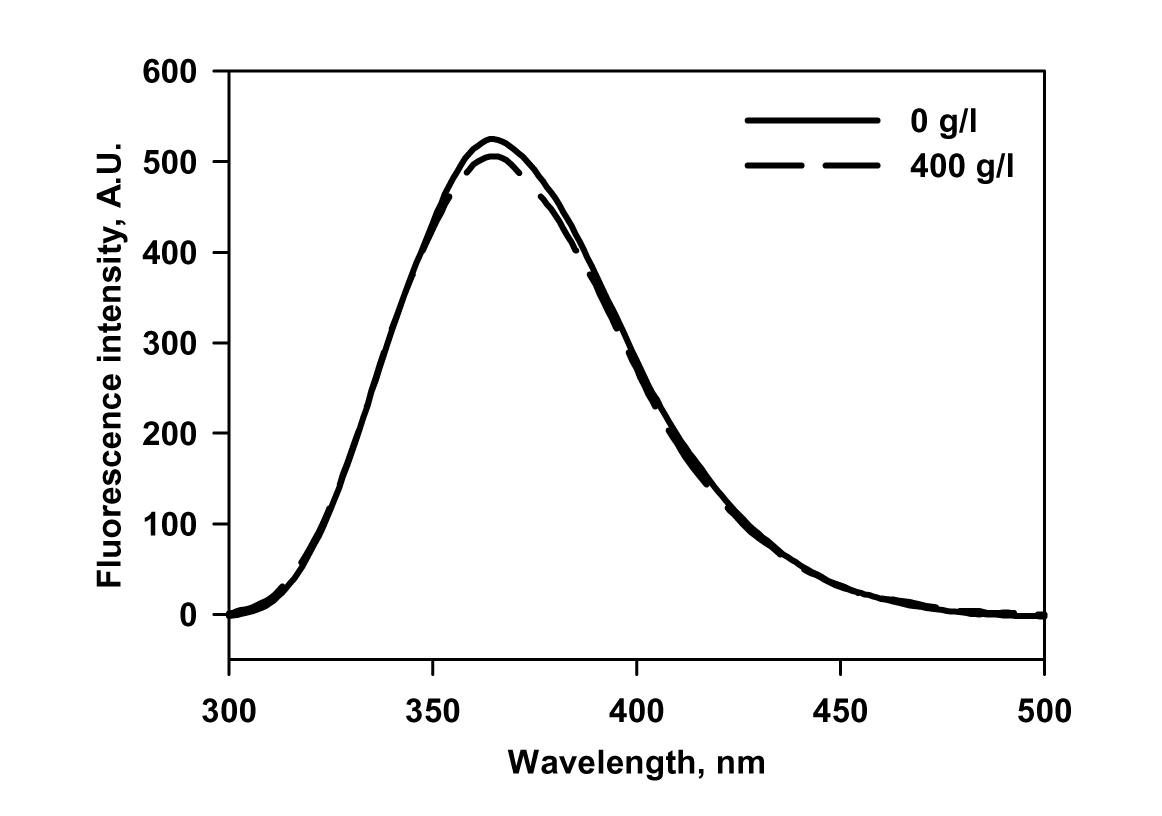

Supplement: Figure S2 — Effect of Ficoll 70 on the fluorescence of NATA. Intrinsic fluorescence of NATA in the absence and presence of 400 g/l Ficoll 70. (TIF) [file pone.0078936.s002.tif]

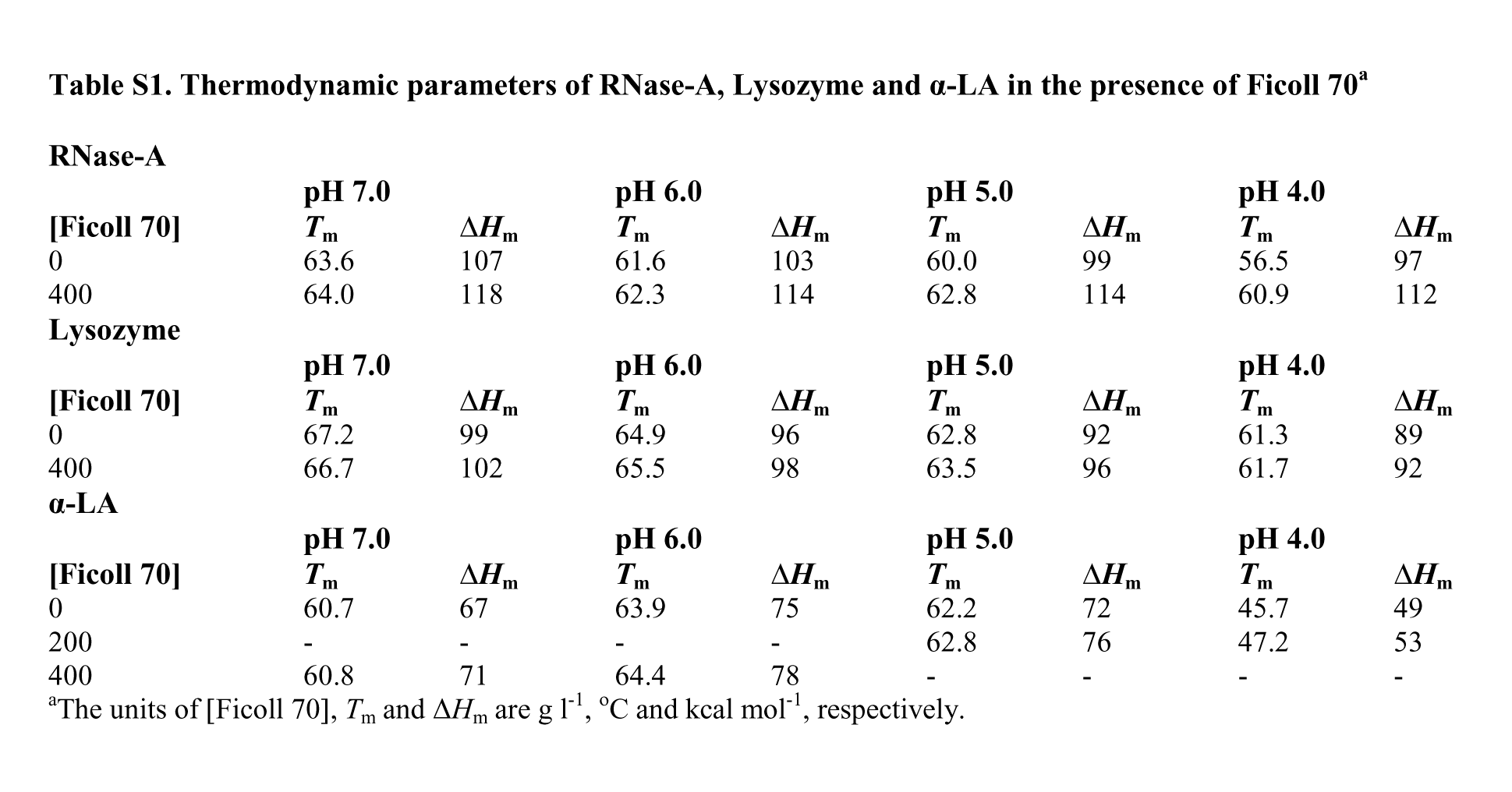

Supplement: Table S1 — (TIF) [file pone.0078936.s003.tif]
